# Heart Fastened to a Dying Animal

**DOI:** 10.3201/eid1808.AC1808

**Published:** 2012-08

**Authors:** Polyxeni Potter

**Affiliations:** Centers for Disease Control and Prevention, Atlanta, Georgia, USA

**Keywords:** art science connection, emerging infectious diseases, art and medicine, Heart fastened to a dying animal, Jules Adler, Transfusion of a Goat’s Blood, transfusion, transplantation, about the cover

**Figure:**
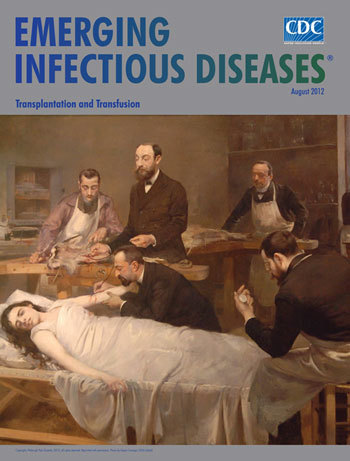
**Jules Adler (1865–1952) *Transfusion of a Goat's Blood* (1892) Oil on canvas (129.5 cm × 195.6 cm)** Copyright, Pittsburgh Post-Gazette, 2010, all rights reserved. Reprinted with permission. Photo by Alyssa Cwanger, 2006

“Medea unsheathed a knife, and cut the old man’s throat, and letting the old blood out, filled the dry veins with the juice. When Aeson had absorbed it, part through his mouth, and part through the wound, the white of his hair and beard quickly vanished, and a dark color took its place. At a stroke his leanness went, and his pallor and dullness of mind. The deep hollows were filled with rounded flesh, and his limbs expanded.” So wrote Ovid in the Metamorphoses about Medea performing a crude transfusion to rejuvenate the father of her beloved Jason. The “barbarian witch” infused a concoction of “dark juices…. the wings and flesh of a vile screech-owl and the slavering foam of a sacrificed werewolf…. the scaly skin of a water-snake, the liver of a long-lived stag….”

This story from antiquity traces human fascination with blood—at first with the loss of blood and its connection with weakness and death, later with blood transference from strong persons or animals to the infirm as therapy, usually by mouth. Interest and reports of attempts at some form of transfusion continued throughout the Middle Ages and up until the 1600s and William Harvey’s description of the human circulatory system, which ushered in a new era of attempts at transfusion. In addition to blood, experimentation involved beer, opium, and milk, infused directly into the veins and arteries of animals, which invariably died.

“What if we transfused the blood of an Archbishop into a Quaker,” joked Samuel Pepys in a 1666 diary entry making light of a hot topic of his day: taking blood from a beast and putting it into another beast or into the veins and arteries of a person. But on a more serious note, he wrote, the practice might “if it takes, be of mighty use to man’s health, for the amending of bad blood by borrowing from a better body.” A fine record of the anxieties of its age, Pepys’ diary documented unfolding historical events, becoming a valued primary source. The first person in England to receive a transfusion from a sheep was “indigent and ‘looked upon as a very freakish and extravagant man’…. About 32 years of age…. He spoke Latin well … but his brain was a little too warm…. They purpose to let in about 12 ounces; which, they compute, is what will be let in in a minute’s time by a watch.”

The sensational nature of early transfusions made them a frequent topic of write-ups, now a substantial historical record. But the subject’s appeal spilled into other areas, art among them. Jules Adler’s *Transfusion of a Goat's Blood*, on this month’s cover, is the pictorial representation of a transfusion that took place in 1890 and the circumstances surrounding it. Adler’s undertaking showed that transfusion was perceived akin to other major historical themes of painting and part of the history of medicine.

The painting was commissioned by respected Paris physician Samuel Bernheim (1855–1915), a tuberculosis specialist who established a charity to send patients and their children to the seaside and other open areas as part of the sanatorium movement. Bernheim was depicted as the central figure standing above the patient. The procedure described involved transfusing some 200 g of blood from the goat to a female patient likely in an effort to strengthen immunity. The same year, Bernheim published the article “Transfusion of Goat’s Blood and Lung Tuberculosis.”

Adler was born in the commune Luxeuil-les-Bains in eastern France. Not much is known about his life, except that his talent was recognized early and his parents moved to Paris where he attended the École Nationale Supérieure des Arts Décoratifs and the École des Beaux-Arts. He also studied at the Académie Julian under William Bouguereau and Tony Robert-Fleury. Even though he is often categorized as an academic painter, Adler is better known as “the painter of the humble” for his affinity to the common people, whose plight he epitomized in his best works. While he moved within Paris Salon circles and espoused academic conventions, he took off on his own to create a naturalist style akin to social realism and attain broad audience appeal. He painted laborers (*The Strike at Creusot*) and the poor (*The Weary*), championed social causes, and addressed in his works anti-Semitism, injustice, and the alienation of modern life.

*Transfusion of a Goat's Blood* was shown in the Salon and won an award, but this success did not affect the popularity of the painting or the artist. The Paris École de Médicine, perhaps not wanting to draw attention to a discredited procedure, relegated the painting to a stairway. By this time, there had been plenty of evidence that human and animal blood were not compatible. But some physicians were using animal serum to treat diphtheria in children, and others wondered if animal fluids might cure various human diseases. In 1901, Karl Landsteiner would identify blood types and their role in safe human-to-human blood transfusions.

Adler’s painting, like other illustrations of medical history, recorded procedures and related conventions through the artist’s lens, which demystified some and chose to romanticize others. On the one hand, instead of a heroic physician, the painting showed a team, suggesting that the faces might change but not the procedure, which was guided by protocol. On the other hand, the depiction of blood was discrete, visible only against the extreme paleness of the patient and against the predominance of white in the room. Long dark hair frames the dying maiden’s face against the white pillow and sheets, her vulnerable situation a stark contrast against that of the medicine men in suits, who seemed to have everything under control. Behind Bernheim, a goat was stretched out on an ordinary bench. The connection between the animal and the human patient was a piece of simple rubber tubing with a cannula at each end.

Attempts at sharing blood and other tissues across species have not abated, though without Medea’s flamboyant concoctions and recipient fear of incurring animal traits or the donor’s religious beliefs. Challenges persist because of transfusion and organ and cell transplant–associated diseases. Demand is high, but the supply of allografts is limited. Immunologic rejection remains a problem. And not the least are infectious disease threats: allograft- and xenograft-derived zoonotic and nonzoonotic infections and infections introduced during tissue processing, the transplant procedure, or post-operative hospitalization.

Threat identification and response related to donor and recipient are improving the process. But problems remain with the specificity and sensitivity of tests, unknown pathogens, and faulty histories. Many zoonotic infections, from hepatitis E and human granulocytic anaplasmosis to solid organ transplant-associated lymphocytic choriomeningitis, can cause severe complications in recipients who, unlike Medea’s charge, are not so fortunate.

A long history of close cohabitation speaks for a far closer connection between animals and humans than shown by the simple rubber tubing in Adler’s painting. This history is celebrated in poetry too, which examines, among other subjects, the interface of their health and common fate—the never-ending calamity of death. W.B. Yeats, pondering his own declining health and weak aging body, was able to see beyond the literal cannula of transfusion. In a precocious “one health” stance, in the poem “Sailing to Byzantium” (1928), he conjured the immense damage to the body from illness and physiologic decline. The spirit, imagination and intellect, which he posed as the only way to remain vital in the face of this decline, also fuel continued medical efforts to improve health and prolong life—literally through transfusion and solid tissue transplants and metaphorically when the perennially young human heart finds itself “fastened to a dying animal,” the body.
